# Modification of the Maxwell–Wagner Heterogeneous Dielectric Model for Heterogeneous Polymers and Emulsions

**DOI:** 10.3390/polym14132743

**Published:** 2022-07-05

**Authors:** Jiangbo Qian, Shimi Yan, Zhenyu Li, Ling Yu, Xinlei Wang, Zhijie Zhang, Junze Sun, Xu Han

**Affiliations:** 1 Department of Power Engineering, North China Electric Power University, Baoding 071003, China; yanshimi@ncepu.edu.cn (S.Y.); lizhenyu@ncepu.edu.cn (Z.L.); yuling@ncepu.edu.cn (L.Y.); wangxinlei@ncepu.edu.cn (X.W.); zhangzhijie@ncepu.edu.cn (Z.Z.); sunjunze@ncepu.edu.cn (J.S.); hanxu@ncepu.edu.cn (X.H.); 2 Hebei Key Laboratory of Low Carbon and High Efficiency Power Generation Technology, North China Electric Power University, Baoding 071003, China

**Keywords:** heterogeneous polymers, emulsions, M–W, integral modification, dielectric constant

## Abstract

In heterogeneous polymers and emulsions, the volume fraction of the discrete phase and the frequency of electromagnetic waves affect the accuracy of the dielectric model. The integral method was used to modify the Maxwell–Wagner (M–W) heterogeneous dielectric theory, and a new model for the complex dielectric constant of polymers and emulsions was established. The experimental data were compared with the results of the M–W heterogeneous dielectric integral modification model and other theoretical models for different frequencies and volume fractions of the discrete phase. We discovered that with a decreasing volume fraction of the discrete phase, the dominant frequency range of the integral modification model expanded. When the volume fraction of the discrete phase is 10%, the dominant frequency range reaches 3 GHz. When the volume fraction of the discrete phase is 1%, the dominant frequency range reaches 4 GHz. When the volume fraction of the discrete phase is 0.06%, the dominant frequency range of the real part reaches 9.6 GHz, and the dominant frequency range of the imaginary part reaches 7.2 GHz. These results verify the advantages of the M–W modification model, which provides a theoretical basis to study the dielectric properties of polymers and emulsions, as well as for microwave measurement.

## 1. Introduction

Heterogeneous polymers and emulsions are widely used in daily life and industrial production. Their physical properties are inextricably linked to the compositional characteristics of the continuous and discrete phases [1,2,3,4,5].

Microwave measurement technology has been widely used in the measurement of the components of polymers and emulsions. Biodiesel is used as an alternative fuel to petro-diesel; the moisture content influences the extent of transesterification of diesel mixture and thus the fuel characteristics [6]. Insulating oil serves in insulation, arc suppression, and cooling in power systems [7,8,9]. The accurate detection of microwater is critical to the safe operation of substation equipment [10,11,12,13,14]. Furthermore, microwave technology is used for subgrade soil moisture detection [15] and steam turbine wet steam measurement [16]. Therefore, research into the dielectric properties of heterogeneous polymers and emulsions has significant practical value.

Many researchers have conducted in-depth studies on the dielectric properties of heterogeneous polymers and emulsions, and they have proposed or modified a number of theoretical models, such as the Lichtenecker logarithmic model, Brown model, CRIM formula, Looyenga model, Bruggeman symmetry model, series-parallel calculation formula, Rayleigh model, Maxwell–Garnett model, and M–W heterogeneous dielectric model. In the measurements of polymeric components using technology, it was discovered that these theoretical models were not satisfactory in cases where the volume fraction of the discrete phase was small. In this work, the M–W heterogeneous dielectric model was modified using the integral method, and a new theoretical model was obtained, which was then verified experimentally.

## 2. Dielectric Model of Two-Phase Heterogeneous Polymers and Emulsions

Currently, the theoretical models applied in studies of the dielectric properties of two-phase heterogeneous polymers and emulsions include the Lichtenecker logarithmic model, Brown model, CRIM formula, Looyenga model, Bruggeman symmetry model, series-parallel calculation formula, Rayleigh model, Maxwell–Garnett model, and M–W heterogeneous dielectric model, the expressions of which are shown in Equations (1)–(10), respectively.

### 2.1. Lichtenecker Logarithmic Model

The Lichtenecker logarithmic model is suitable for calculating the dielectric constant of multiphase mixtures [17]. When the components in the mixture are isotropic, symmetrical, and uniform, the error obtained using this formula is small. It can be expressed as:

(1) $$\ln\varepsilon_{m}=(1-\varphi )\ln\varepsilon_{1}+\varphi \ln\varepsilon_{2}\;$$

In this model, subscripts m, 1, and 2 represent the mixture, continuous phase, and discrete phase, respectively. Here, *ε*_m_ is the dielectric constant of the mixture, *ε*_1_ is the dielectric constant of the continuous phase, *ε*_2_ is the dielectric constant of the discrete phase, and *φ* is the volume fraction of the discrete phase.

### 2.2. Lichtenecker–Rother (L–R) Equation

The L-R model is one of the classical dielectric models [18,19]. Its basic form is shown in Equation (2):

(2) $${\varepsilon_{m}}^{c}=(1-\varphi ){\varepsilon_{1}}^{c}+\varphi {\varepsilon_{2}}^{c}\;$$

In this equation, parameter *c* assumes a value between −1 and 1. Taking different values of parameter *c* in the L–R equation, the L–R equation can be expressed as various forms of the dielectric constant model.

(1) For *c* = 1, it is called the Brown model, which is usually called the linear model:

(3) $$\varepsilon_{m}=(1-\varphi )\varepsilon_{1}+\varphi \varepsilon_{2}$$

(2) For *c* = 0.5, it is called the complex refractive index model (CRIM formula), which is commonly known as the root mean square model:

(4) $$\sqrt{\varepsilon_{m}}=(1-\varphi )\sqrt{\varepsilon_{1}}+\varphi \sqrt{\varepsilon_{2}}$$

The complex refractive index is a model that can be applied to two kinds of materials: a liquid with low to medium viscosity and a multiphase composite medium that contains rough particles with medium coarseness.

(3) For *c* = 1/3, it is called the Looyenga model, which is also known as the cube root model. Looyenga proposed a Looyenga model with a weight factor of 1/3:

(5) $$\varepsilon_{m}^{1/3}=(1-\varphi )\varepsilon_{1}^{1/3}+\varphi \varepsilon_{2}^{1/3}$$

The cubic root model is widely used in the study of the dielectric properties of powder and porous systems in the field of petroleum exploration. Tuncer proposed the Looyenga model for composites with self-similar fractal properties, such as colloidal aggregates and porous materials.

### 2.3. Bruggeman Symmetric Model

The effective medium theory is a mathematical and theoretical model for describing the composite materials’ macroscopic properties. This theory obtains composite material properties by averaging the properties of various components in composite materials. Based on this theory, Bruggeman proposed a new mixing rule [20]:

(6) $$\varphi \frac{\varepsilon_{2}-\varepsilon_{m}}{\varepsilon_{2}+2\varepsilon_{m}}+(1-\varphi )\frac{\varepsilon_{1}-\varepsilon_{m}}{\varepsilon_{1}+2\varepsilon_{m}}=0$$

The Bruggeman symmetry model is applied as follows: under static field conditions, only dipole interactions between the particles are considered, localization is not considered, and the particles are spherical and tend to be of average size. There are only two phases in the composite, and the particles are filled with a matrix medium.

### 2.4. Series-Parallel Calculation Formula

Based on the study of oil and water polarity molecules, Huang zhenghua [21] proposed the series-parallel calculation formula. It can be expressed as:

(7) $$\varepsilon_{m}=k[(1-\varphi )\varepsilon_{1}+\varphi \varepsilon_{2}]+(1-k)\varepsilon_{1}\varepsilon_{2}{\left[(1-\varphi )\varepsilon_{2}+\varphi \varepsilon_{1}\right]}^{-1}\;$$

where *k* = 2*φ*(5 − 3*φ*)^−1^.

### 2.5. Rayleigh Model

For a nonuniform dielectric composed of two phases, if the relative dielectric constants of the two phases are *ε*_1_ and *ε*_2_, and the volume fraction for the phase with the dielectric constant *ε*_1_ is *φ*, the following theoretical formula is obtained [19]:

(8) $$\frac{\varepsilon_{m}-1}{\varepsilon_{m}+2}=(1-\varphi )\frac{\varepsilon_{1}-1}{\varepsilon_{1}+2}+\varphi \frac{\varepsilon_{2}-1}{\varepsilon_{2}+2}$$

The model assumes that the dispersed particles in the mixture are homogeneous spherules.

### 2.6. Maxwell–Garnett Model

The Maxwell–Garnett model [22] is evolved from the M–W model, as shown in Equation (9):

(9) $$\varepsilon_{m}=\varepsilon_{2}\left[\frac{\varepsilon_{1}+2\varepsilon_{2}+2(1-\varphi )(\varepsilon_{1}-\varepsilon_{2})}{\varepsilon_{1}+2\varepsilon_{2}-(1-\varphi )(\varepsilon_{1}-\varepsilon_{2})})\right]\;$$

The theoretical method is based on the physical model of spherical particles in a matrix material, which is suitable for particle dispersion systems.

### 2.7. M–W Heterogeneous Dielectric Model

For anisotropic dielectric materials, since each part has different dielectric coefficients and conductivities, they show different electrical properties. Under an applied external electric field, charge accumulation will occur inside the medium, which is known as the M–W effect. The dielectric model based on this effect is given by [23]:

(10) $$\frac{\varepsilon_{m}-\varepsilon_{1}}{\varepsilon_{m}+2\varepsilon_{1}}=\varphi \frac{\varepsilon_{2}-\varepsilon_{1}}{\varepsilon_{2}+2\varepsilon_{1}}$$

In this paper, the Lichtenecker logarithmic model, Rayleigh model, Bruggeman symmetric model, series-parallel calculation formula, and M–W heterogeneous dielectric model, which are widely used in dielectric property research, are selected. The experimental results were compared with the calculated values of the selected dielectric models and the M–W heterogeneous dielectric integral modification model.

## 3. M–W Heterogeneous Dielectric Model and Its Integral Modification

### 3.1. M–W Basic Model

For anisotropic dielectric materials, each component has different dielectric coefficients and conductivities so that the component also shows different electrical properties. Under the application of an external electric field, charge accumulation will occur inside the medium, which is known as the M–W effect. When the temperature is constant, the dielectric constant of the electrolyte is constant and real in the electrostatic field and is complex in the alternating electric field. The real part of the medium’s complex dielectric constant is close to a constant in the low-frequency band and is roughly equal to the static permittivity of the medium. In the high-frequency band, when the frequency of the alternating electric field increases, the real and imaginary parts of the medium’s complex dielectric constant vary with frequency.

**Figure 1:**
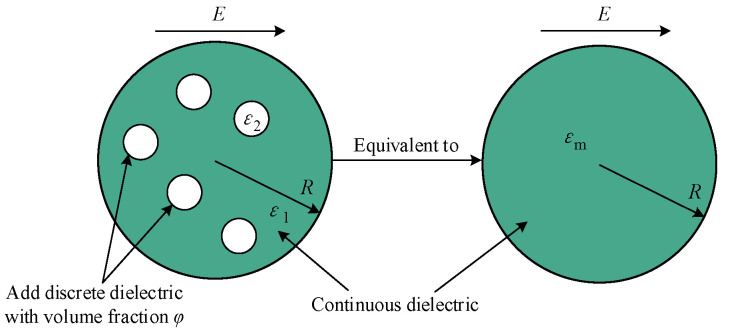
M–W dielectric model.

Maxwell was the first person to derive the theory of the electric field [24,25]. The composition of a heterogeneous material is as follows: many dielectric spheres (micro-/nanodiameter) with dielectric constant *ε*_2_ are uniformly distributed in a continuous medium with dielectric constant *ε*_1_ and volume fraction *φ*, as shown in Figure 1.

Maxwell used two steps to deduce the theory: (1) solve the Laplace equation to calculate the electric potential of a spherical particle in the medium and calculate the electric potential *E* of a large sphere containing N such particles (assuming that the concentration of the small spheres in the large sphere is very low, and thus ignoring the interaction between the spheres); (2) treat this large heterogeneous sphere containing N small spherical particles as a homogeneous sphere with an equivalent dielectric constant *ε*_m_, and its external potential is equal to *E*. However, in the initial derivation of the mixing theory, Maxwell used the static dielectric constant for particles and continuous media. Later, Wagner developed Maxwell’s theoretical method, replaced the static dielectric constant in the Maxwell mixing equation with the complex dielectric constant, and obtained the famous M–W equation given in Equation (10).

### 3.2. Integral Modification of the M–W Dielectric Model

**Figure 2:**
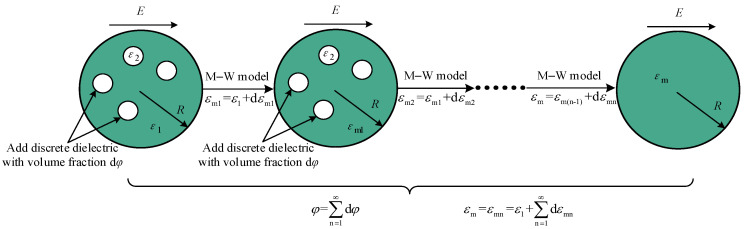
Integral principle diagram.

Based on the M–W dielectric model, the integral method is used to treat the model. As shown in Figure 2, the volume fraction *φ* of the discrete medium in the continuous medium is gradually added to the continuous medium with the infinitesimal d*φ*. Every time, the complex permittivity is calculated using the M–W model until the volume fraction of the discrete phase increases to *φ*. Finally, the heterogeneous polymers’ equivalent complex permittivity is obtained.

Since the duty ratio of the discrete phase is very small, the dielectric constant of the heterogeneous polymers is almost infinitely close to that of the continuous phase; that is, *φ* → d*φ*, then *ε*_1_ → *ε*_m_. Equation (10) is then transformed into a differential form:

(11) $$\frac{d\varepsilon_{m}}{3\varepsilon_{m}}=\frac{\varepsilon_{2}-\varepsilon_{m}}{\varepsilon_{2}+2\varepsilon_{m}}d\varphi$$

The left-hand and right-hand sides of the above equation are subjected to identity transformation to obtain:

(12) $$\frac{2\varepsilon_{m}+\varepsilon_{2}}{3\varepsilon_{m}\left(\varepsilon_{m}-\varepsilon_{2}\right)}d\varepsilon_{m}=-d\varphi$$

By splitting the left-hand side of the above equation into the form of the sum of two fractions, the following can be obtained:

(13) $$\left[\frac{2}{3}\frac{1}{\varepsilon_{m}-\varepsilon_{2}}+\frac{\varepsilon_{2}}{3}\frac{1}{{\varepsilon_{m}}^{2}-\varepsilon_{2}\varepsilon_{m}}\right]d\varepsilon_{m}=-d\varphi$$

The differential process is integrated; that is, the antiderivative of both ends of Equation (13) is obtained:

(14) $$\frac{2}{3}\int \frac{d\varepsilon_{m}}{\varepsilon_{m}-\varepsilon_{2}}+\frac{\varepsilon_{2}}{3}\int \frac{d\varepsilon_{m}}{{\varepsilon_{m}}^{2}-\varepsilon_{2}\varepsilon_{m}}=-\int d\varphi$$

Then, we obtain:

(15) $$\frac{2}{3}\ln\left(\varepsilon_{2}-\varepsilon_{m}\right)+\frac{1}{3}\ln\left(\frac{\varepsilon_{2}-\varepsilon_{m}}{\varepsilon_{m}}\right)=-\varphi +A$$

where *φ* = 0–1. Considering the special case when *φ* = 0, there is no discrete phase in the heterogeneous polymers, and the complex dielectric constant of the polymers should be equal to that of the continuous phase (i.e., *ε*_m_ = *ε*_1_). When *φ* = 0, the boundary condition of *ε*_m_ = *ε*_1_ can be combined with Equation (15) to obtain:

(16) $$A=\frac{2}{3}\ln\left(\varepsilon_{2}-\varepsilon_{1}\right)+\frac{1}{3}\ln\left(\frac{\varepsilon_{2}-\varepsilon_{1}}{\varepsilon_{1}}\right)$$

Substituting the above formula for A into Equation (15) yields:

(17) $$\ln\left(\frac{\varepsilon_{m}}{\varepsilon_{1}}\right)=3\left[\varphi -\ln\left(\frac{\varepsilon_{2}-\varepsilon_{1}}{\varepsilon_{2}-\varepsilon_{m}}\right)\right]$$

By taking the exponent of *e* on both sides of the above equation, the iterative expression of the equivalent permittivity of the heterogeneous dielectric can be obtained:

(18) $$\varepsilon_{m}=\varepsilon_{1}{\left(\frac{\varepsilon_{2}-\varepsilon_{m}}{\varepsilon_{2}-\varepsilon_{1}}\right)}^{3}e^{3\varphi }$$

The above iterative equation has limitations in solution accuracy because of the repeated iterations. The iterative formula is expanded to obtain:

(19) $${\varepsilon_{m}}^{3}-3\varepsilon_{1}{\varepsilon_{m}}^{2}+\left[3{\varepsilon_{2}}^{2}+\frac{{\left(\varepsilon_{2}-\varepsilon_{1}\right)}^{3}}{\varepsilon_{1}e^{3\varphi }}\right]\varepsilon_{m}-{\varepsilon_{2}}^{3}=0$$

The complex coefficient unary cubic equation shown in Equation (19) should be solved according to Cardan’s solution formula [26]; let a=1, $b=-3\varepsilon_{2}$, $c=3{\varepsilon_{2}}^{2}+\frac{{\left(\varepsilon_{2}-\varepsilon_{1}\right)}^{3}}{\varepsilon_{1}e^{3\varphi }}$, $d=-{\varepsilon_{2}}^{3}$, $v=\frac{{\left(3\times (4ac^{3}-b^{2}c^{2}-18abcd+27a^{2}d^{2}+4b^{3}d)\right)}^{0.5}}{18a^{2}}$, $u=\frac{9abc-27a^{2}d-2b^{3}}{54a^{3}}$.

If |u+v|<|u−v|, then $\Gamma ={\left(u-v\right)}^{\frac{1}{3}}$; otherwise, $\Gamma ={\left(u+v\right)}^{\frac{1}{3}}$. When the value of *Γ* is determined, *Π* is determined.

If |u+v|<|u−v|, then Π=0; otherwise, $\Pi =\frac{\left(b^{2}-3ac\right)}{9a\Gamma }$. After the values of the above parameters are obtained, the solution is:

$$\varepsilon_{m1}=\Gamma +\Pi -\frac{b}{3a}$$

$$\varepsilon_{m2}=w\Gamma +w^{2}\Pi -\frac{b}{3a}$$

$$\varepsilon_{m3}=w^{2}\Gamma +w\Pi -\frac{b}{3a}$$

where $w=\frac{\left(-1+i\sqrt{3}\right)}{2}$.

Considering these three solutions for heterogeneous polymers and emulsions, we found the following laws through simulation calculations. When the continuous phase has a smaller dielectric constant than the discrete phase, *ε*_m3_ gives the correct value. When the continuous phase has a greater dielectric constant than the discrete phase, *ε*_m1_ gives the correct value. When they have similar dielectric constants, *ε*_m2_ gives the correct value.

## 4. Dielectric Properties Experiment

To validate the accuracy of the M–W heterogeneous dielectric integral modification model, dielectric properties must be measured experimentally, and experimental data must be compared with the results of the M–W heterogeneous dielectric integral modification model and other theoretical models.

The experimental object was a polymer emulsion of Karamay 25 insulating oil and deionized water. The insulating oil was the continuous phase, and deionized water was the discrete phase.

### 4.1. Experimental Procedure and Experimental Equipment

**Figure 3:**
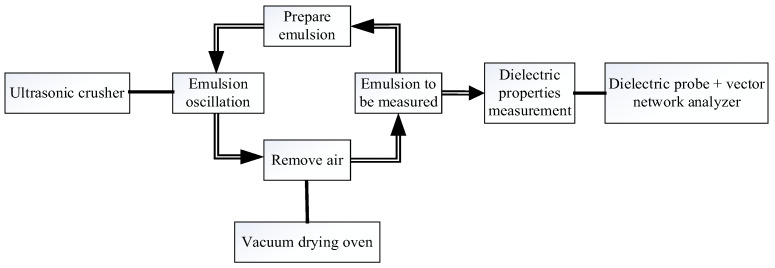
Experimental flowchart.

In this experiment, the volume fraction of deionized water (*φ*) increased from 0% to 10%. The insulating oil and deionized water were mixed into a mixed system. Then, the mixture was stirred uniformly. First, the uniformly mixed oil–water emulsion was ultrasonicated. After sufficient ultrasonication, the emulsion was treated in a vacuum drying oven to remove the small bubbles in the emulsion. Then, the dielectric constant of the polymer emulsion was measured. The actual process is shown in Figure 3.

A vector network analyzer (E5071C) with an emulsion dielectric test probe (N1501A) and an ultrasonic crusher are the main equipment used in this experiment. The vector network analyzer uses a probe to measure the complex dielectric constant of a polymer emulsion. The ultrasonic crusher is used to shock the oil–water polymer emulsion by ultrasonic waves in order to fully mix the emulsion.

### 4.2. Experimental Measurement

**Figure 4:**
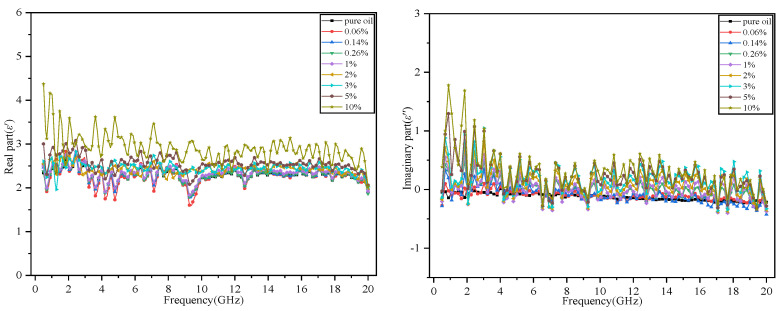
Measurement diagram of dielectric properties (23.5 °C at 1 atm).

The experimental temperature was maintained at 23.5 °C, and the pressure was maintained at 1 atm. The measuring frequency range of the instrument was set to 500 MHz–20 GHz, and a vector network analyzer with a dielectric probe was used to measure the complex dielectric constant of the polymer emulsion under different moisture contents. The measurement results are shown in Figure 4.

Figure 4 shows that the real part of the dielectric constant decreases with increasing frequency and increases with increasing moisture content. Regarding the imaginary part of the dielectric constant, because insulating oil has a low dielectric loss due to its stability, the imaginary part of the dielectric constant of the pure oil is close to zero. The dielectric loss increases with increasing moisture content, and its imaginary part deviates from zero.

*ε*_0_, *ε*_∞_ and *τ* of the pure oil can be obtained by numerically fitting the experimental values in Figure 4 to the formula for the dielectric constant given in Equation (20) [27].

(20) $$\varepsilon^{\prime }=\varepsilon_{\infty }+\frac{\varepsilon_{0}-\varepsilon_{\infty }}{1+{\left(\omega \tau \right)}^{2}};\;\varepsilon^{\prime \prime }=\frac{\left(\varepsilon_{0}-\varepsilon_{\infty }\right)\omega \tau }{1+{\left(\omega \tau \right)}^{2}}$$

where *ε*_0_ is the static dielectric constant of the medium, *ε*_∞_ is the optical frequency dielectric constant of the medium, *ω* is the angular frequency of the alternating electromagnetic field, and *τ* is the dielectric relaxation time of the medium.

The related dielectric characteristic parameters of pure oil and pure water [28,29,30,31] are shown in Table 1.

Similarly, numerical fitting can be used to obtain the static dielectric constant *ε*_0_, optical frequency dielectric constant *ε*_∞_, and relaxation time *τ* of the oil–water polymer emulsions with different moisture contents. Then, the smooth distribution curves of the experimental values with frequency under different moisture contents can be obtained.

## 5. Results and Discussion

### 5.1. Analysis of the Influence of Frequency on Dielectric Properties

**Figure 5:**
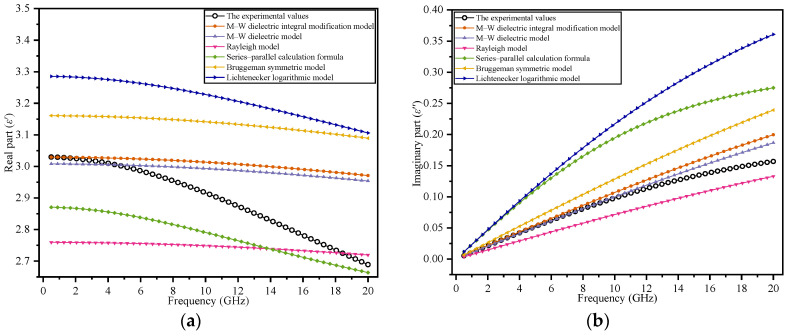

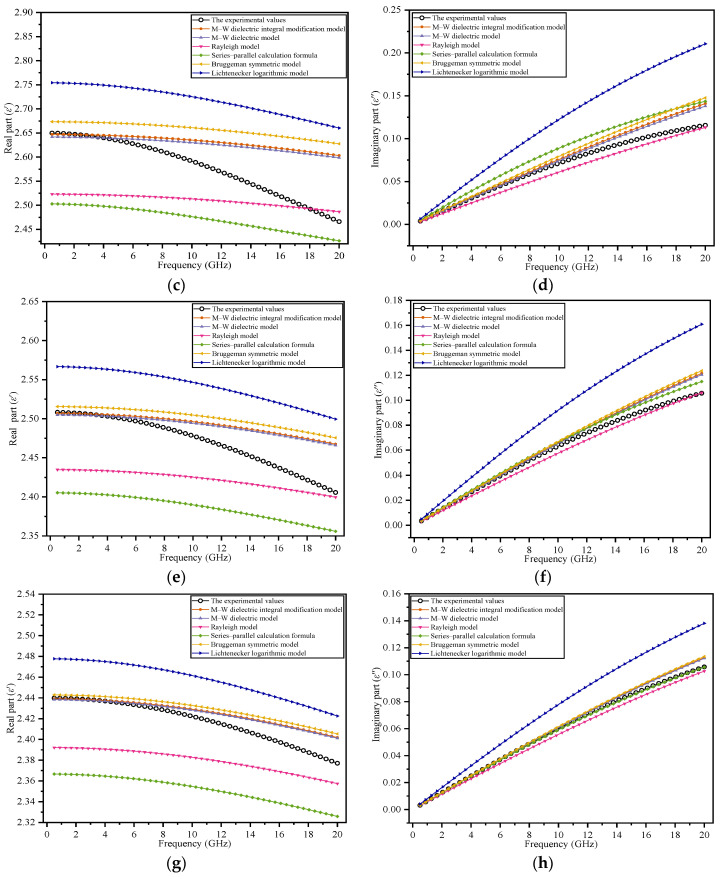
Comparison between theoretical models and experimental values under multiple moisture contents: (**a**,**b**) *φ* = 10%; (**c**,**d**) *φ* = 5%; (**e**,**f**) *φ* = 3%; (**g**,**h**) *φ* = 2%.

In practical industrial applications, the measurement frequency is mostly in the centimeter band, and therefore, this band range is chosen to analyze the dielectric properties of the theoretical model. The complex dielectric constants of pure oil and pure water at different frequencies were calculated using the values of the dielectric properties of pure oil and pure water in Table 1 and Equation (20). Thus, the smooth distribution curves in the cm band of the different theoretical models under different moisture contents were plotted. Figure 5 shows smooth distribution curves for the different theoretical models and the experimental values.

In Figure 5a,b, the values obtained using the M–W dielectric integral modification model are the closest to the experimental values (for frequencies less than 3 GHz); however, for the frequencies greater than 3 GHz, the M–W dielectric integral modification model is no longer advantageous compared with other models.

Figure 5c,d shows that when the moisture content decreases to 5%, the dominant frequency range of the M–W dielectric integral modification model expands to 3.2 GHz (the dominant frequency range of the M–W dielectric integral modification model is defined within 3.2 GHz at *φ* = 5%).

From Figure 5e–h, it is observed that when the moisture content decreases, the dominant frequency range of the M–W dielectric integral modification model clearly expands for both the real and imaginary parts of the dielectric constants.

**Figure 6:**
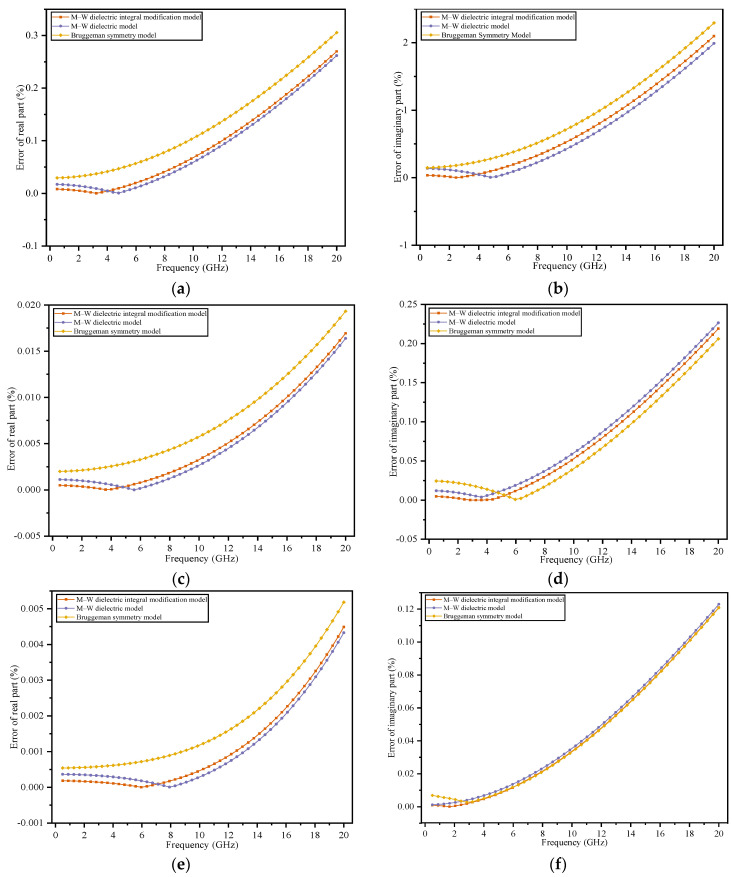

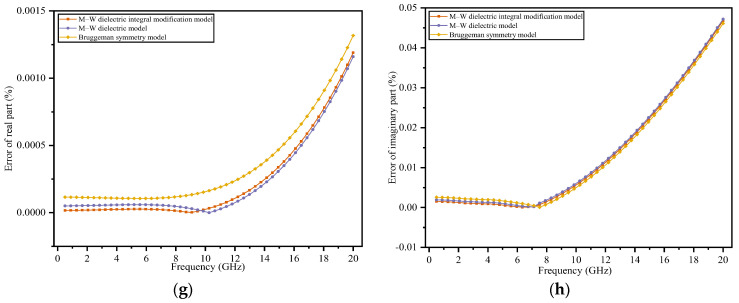
Error comparison of different models under multiple moisture contents. When the moisture content is 1% or less, the Lichtenecker logarithmic model, Rayleigh model, and series-parallel calculation formula have significantly higher errors than the Bruggeman symmetric model, so the error is omitted: (**a**,**b**) *φ* = 1%; (**c**,**d**) *φ* = 0.26%; (**e**,**f**) *φ* = 0.14%; (**g**,**h**) *φ* = 0.06%.

When the moisture content is less than 1%, the data show that the difference between the models is small, so error analysis is used to more clearly elucidate the differences among the dielectric models. Error comparisons for moisture contents of 1% and below 1% are shown in Figure 6.

Figure 6g,h shows that when the moisture content is 0.06%, the dominant frequency range of the real part extends to 9.6 GHz, and the dominant frequency range of the imaginary part extends to 7.2 GHz; that is, the modification model performance improves compared with that at a higher moisture content.

Figure 6 shows that when the moisture content decreases, the dominant frequency range of the M–W dielectric integral modification model widens, indicating that the accuracy-dominant frequency range of the M–W integral modification model is directly related to the volume fraction of the discrete phase. The absolute error in the dominant frequency range decreases with decreasing moisture content.

Generally, for the moisture content in the 5–10% range, the dominant frequency range of the M–W dielectric integral modification model reaches 3.2 GHz. If the frequency exceeds 3.2 GHz at this moisture content, the error of the M–W dielectric integral modification model will increase and will be higher than those of the other theoretical models. When the moisture content is below 5%, the dominant frequency range of the M–W dielectric integral modification model is wider (reaching beyond 3.2 GHz), and the error is smaller. Thus, the M–W dielectric integral modification model has higher accuracy at a small volume fraction of the discrete phase and at low frequencies.

**Figure 7:**
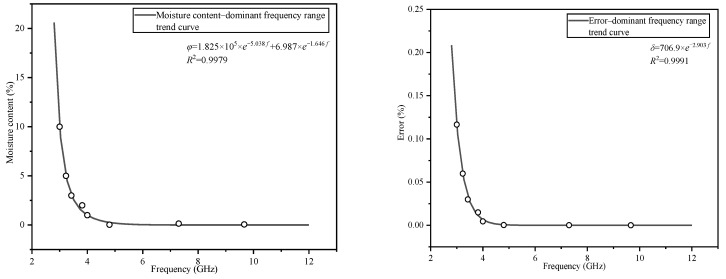
Trend graph of the real part of the dielectric constant.

**Figure 8:**
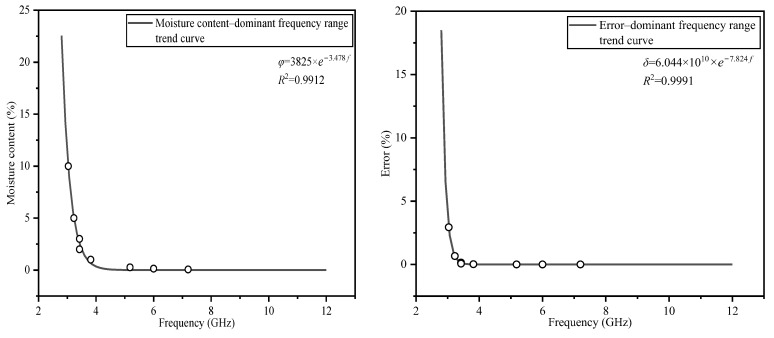
Trend graph of the imaginary part of the dielectric constant.

Figure 7 and Figure 8 show the fitting trend diagrams of the real and the imaginary parts regarding the relationship between the moisture content and the dominant frequency range, corresponding to the error and the dominant frequency range, respectively.

The trend plots presented in Figure 7 and Figure 8 show that when the moisture content increases, the dominant frequency range of the M–W dielectric integral modification model decreases, and the relative error increases when the dominant frequency range decreases. The trend diagrams show that when the moisture content decreases, the dominant frequency range of the M–W dielectric integral modification model expands more rapidly. When the volume fraction of the discrete phase is 10%, the dominant frequency range reaches 3 GHz. When the volume fraction of the discrete phase is 1%, the dominant frequency range reaches 4 GHz. When the volume fraction of the discrete phase is 0.06%, the dominant frequency ranges of the real and imaginary parts reach 9.6 and 7.2 GHz, respectively.

The M–W dielectric integral modification model will no longer be advantageous compared with the other models in the region above the fitting curve. This result clarifies the statement that the accuracy of the M–W dielectric integral modification model is related to the volume fraction of the discrete phase.

### 5.2. Analysis of the Influence of Moisture Content on the Dielectric Properties

Centimeter band analysis reflects the effect of frequency on the accuracy of dielectric models. However, at a certain frequency, the effect of the volume fraction of the discrete phase on the accuracy of dielectric models is not reflected. Therefore, taking three calculation frequencies, *f*_1_ = 0.5 GHz, *f*_2_ = 2 GHz, and *f*_3_ = 3 GHz, the real and imaginary parts of the dielectric model under different moisture contents at a certain frequency were calculated using the calculated frequency value *f*. Then, the calculated results were compared with the experimental values to more comprehensively compare the accuracy of different theoretical models.

**Figure 9:**
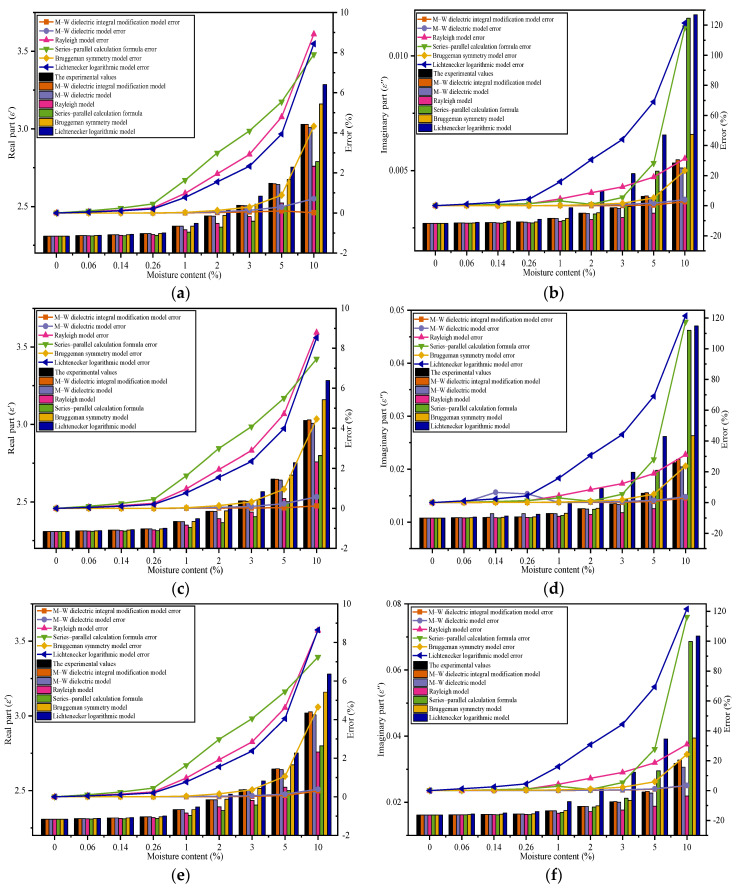
Comparison of different models at multiple frequencies: (**a**,**b**) *f*_1_ = 0.5 GHz; (**c**,**d**) *f*_2_ = 2 GHz; (**e**,**f**) *f*_3_ = 3 GHz.

Figure 9 compares the calculated results of different dielectric models and the experimental values.

According to Figure 9, for a given frequency, the error increases with increasing moisture content for all theoretical models. The M–W dielectric integral modification model has the slowest error growth trend and the smallest average error. The error of all theoretical models reaches its maximum value for a moisture content of 10%.

According to the comparison of these three frequencies, in the real part, the average errors of the M–W dielectric integral modification model, Bruggeman symmetry model, and Lichtenecker logarithmic model increase with increasing frequency, while the average errors of the M–W dielectric model, Rayleigh model, and series-parallel calculation formula decrease. For the imaginary part of the dielectric constant, the average errors of the M–W dielectric integral modification model, M–W dielectric model, Bruggeman symmetry model, and Lichtenecker logarithmic model increase with increasing frequency, while the average errors of the Rayleigh model and series-parallel calculation formula decrease.

The error of the M–W dielectric integral modification model decreases with increasing frequency. The analysis of dielectric properties in the centimeter band shows that once the frequency exceeds 3 GHz, the error of the integral modification model in the above diagram will exceed those of the other models, and the curves intersect. The larger the frequency value is, the lower the moisture content corresponding to the intersection is.

## 6. Conclusions

The M–W heterogeneous dielectric model was modified by integration to obtain a new theoretical model. We performed dielectric property measurements on an oil–water polymer emulsion. The experimental temperature was 23.5 °C, the measurement frequency range was 500 MHz–20 GHz, and the pressure was 1 atm. Based on the experiments, the analytical results of the M–W heterogeneous dielectric integral modification model are as follows:

1. Based on the results of the measurements of the complex dielectric constant of the emulsion, the accuracy advantage of the M–W integral modification model becomes more pronounced with a smaller volume fraction of the discrete phase or with lower frequency. By contrast, the accuracy advantage of the integral modification model will be weakened for a larger volume fraction of the discrete phase or higher frequency.
2. When the volume fraction of the discrete phase is 5–10%, for the frequencies lower than 3.2 GHz, the M–W integral modification model has the highest accuracy compared with the other theoretical models. When the volume fraction of the discrete phase is less than 5%, the dominant frequency range of this integral modification model exceeds 3.2 GHz. When the volume fraction of the discrete phase is 0.06%, the dominant frequency ranges of the real and imaginary parts reach 9.6 and 7.2 GHz, respectively.
3. Regarding the error, a lower volume fraction of the discrete phase corresponds to a smaller error of the modified model in the dominant frequency range. All theoretical models have much larger errors for the imaginary part of the dielectric constant than for the real part, indicating that these dielectric models are more useful for the real part.
4. The M–W heterogeneous dielectric integral modification model effectively improves the accuracy of the dielectric constant calculation for heterogeneous polymers and emulsions. This integral modification model can be used as a theoretical basis to study the dielectric properties of polymers and emulsions, as well as for microwave physical property measurements.

## Figures and Tables

**Table 1:** Dielectric property value (23.5 °C, at 1 atm).

Name of Substance	Static Permittivity *ε*_0_	Optical Frequency Dielectric Constant *ε*_∞_	Relaxation Time *τ*
Deionized water	78.55	2.5	8.35 ps
Karamay 25 insulating oil	2.309	2	2.782 ps

## Data Availability

The data presented in this study are contained within this article.

## References

[1] Nioua Y., El Bouazzaoui S., Achour M.E., Costa L.C. (2017). Modeling microwave dielectric properties of polymer composites using the interphase approach. J. Electromagn. Waves Appl..

[2] Shao J., Zhou L., Chen Y.Q., Liu X., Ji M.B. (2022). Model-based dielectric constant estimation of polymeric nanocomposite. Polymers.

[3] Chen H.L., Xu Y.D., Liu M.Q., Li T. (2021). An experimental study on the dielectric properties of rubber materials. Polymers.

[4] Wang W., Alexandridis P. (2016). Composite polymer electrolytes: Nanoparticles affect structure and properties. Polymers.

[5] Nakajima H., Dijkstra P., Loos K. (2017). The recent developments in biobased polymers toward general and engineering applications: Polymers that are upgraded from biodegradable polymers, analogous to petroleum-derived polymers, and newly developed. Polymers.

[6] Lin C.Y., Ma L. (2022). Effects of water removal from palm oil reactant by electrolysis on the fuel properties of biodiesel. Processes.

[7] Wang H.Q., Cheng L., Cheng Z.D., Yin J., Yang L.J., Liao R.J. (2020). Characterization of water-participant hydrogen bonds in oil-paper insulation investigated with terahertz dielectric spectroscopy. IEEE Trans. Dielectr. Electr. Insul..

[8] Mehmood M.A., Nazir M.T., Li J., Wang F., Azam M.M. (2020). Comprehensive investigation on service aged power transformer insulating oil after decades of effective performance in field. Arab. J. Sci. Eng..

[9] Baird P.J., Herman H., Stevens G.C., Jarman P.N. (2006). Spectroscopic measurement and analysis of water and oil in transformer insulating paper. IEEE Trans. Dielectr. Electr. Insul..

[10] Saha T.K., Purkait P. (2008). Understanding the impacts of moisture and thermal ageing on transformer’s insulation by dielectric response and molecular weight measurements. IEEE Trans. Dielectr. Electr. Insul..

[11] Abdi S., Harid N., Safiddine L., Boubakeur A., Haddad A.M. (2021). The correlation of transformer oil electrical properties with water content using a regression approach. Energies.

[12] Volkov M., Turanova O., Turanov A. (2018). Determination of moisture content of insulating oil by NMR method with selective pulses. IEEE Trans. Dielectr. Electr. Insul..

[13] Oshima S., Nakamura H., Kobayashi H. (2021). Estimation of degradation indices of oil-filled transformer based on the color data of insulating oil. IEEJ Trans. Electr. Electron. Eng..

[14] Kondalkar V.V., Ryu G., Lee Y., Lee K. (2019). Development of highly sensitive and stable humidity sensor for real-time monitoring of dissolved moisture in transformer-insulating oil. Sens. Actuators B.

[15] Mohan R.R., Paul B., Mridula S., Mohanan P. (2015). Measurement of soil moisture content at microwave frequencies. Procedia Comput. Sci..

[16] Qian J.B., Li H.F., Han Z.H., Wu W.M., Yuan D. (2013). Influence of dielectric deposition on the steam wetness measurement. Appl. Mech. Mater..

[17] Goncharenko A.V., Lozovski V.Z., Venger E.F. (2000). Lichtenecker’s equation: Applicability and limitations. Opt. Commun..

[18] Brovelli A., Cassiani G. (2008). Effective permittivity of porous media: A critical analysis of the complex refractive index model. Geophys. Prospect..

[19] Zhong Y., Wang Y.L., Zhang B., Li X.L., Li S.T., Zhong Y.M., Hao M.M., Gao Y.L. (2020). Prediction model of asphalt content of asphalt mixture based on dielectric properties. Adv. Civ. Eng..

[20] Mansoorifar A., Ghosh A., Sabuncu A.C., Beskok A. (2017). Accuracy of the maxwell–wagner and the bruggeman–hanai mixture models for single cell dielectric spectroscopy. IET Nanobiotechnol..

[21] Huang Z.H. (2000). Study on relative dielectric constant of oil-water mixture medium. Surf. Eng. Oil Gas Fields.

[22] Ruppin R. (2000). Evaluation of extended maxwell-garnett theories. Opt. Commun..

[23] Qian J.B., Gu Q.F., Yao H., Zeng W. (2019). Dielectric properties of wet steam based on a double relaxation time model. Eur. Phys. J. E.

[24] Maxwell J.C. (1891). Treatise on Electricity and Magnetism.

[25] Takashima S. (1989). Electrical Properties of Biopolymers and Membranes.

[26] Bardell N.S. (2016). Cubic polynomials with real or complex coefficients: The full picture. Aust. Sen. Math. J..

[27] Debye P. (1929). Polar Molecules.

[28] Nagai M., Yada H., Arikawa T., Tanaka K. (2007). Terahertz time-domain attenuated total reflection spectroscopy in water and biological solution. Int. J. Infrared Millim. Waves.

[29] Uematsu M., Frank E.U. (1980). Static dielectric constant of water and steam. J. Phys. Chem. Ref. Data.

[30] Ro Nne C., Thrane L., Astrand P., Wallqvist A., Mikkelsen K.V., Keiding S.R.R. (1997). Investigation of the temperature dependence of dielectric relaxation in liquid water by THz reflection spectroscopy and molecular dynamics simulation. J. Chem. Phys..

[31] Ronne C., Keiding S.R. (2002). Low frequency spectroscopy of liquid water using THz-time domain spectroscopy. J. Mol. Liq..

